# Supplementary figures and images for: Two novel genes identified by large-scale transcriptomic analysis are essential for biofilm and rugose colony development of Vibrio vulnificus
Source: PLoS Pathog. 2023 Jan 19;19(1):e1011064. doi: 10.1371/journal.ppat.1011064 (PMC9888727; doi:10.1371/journal.ppat.1011064)

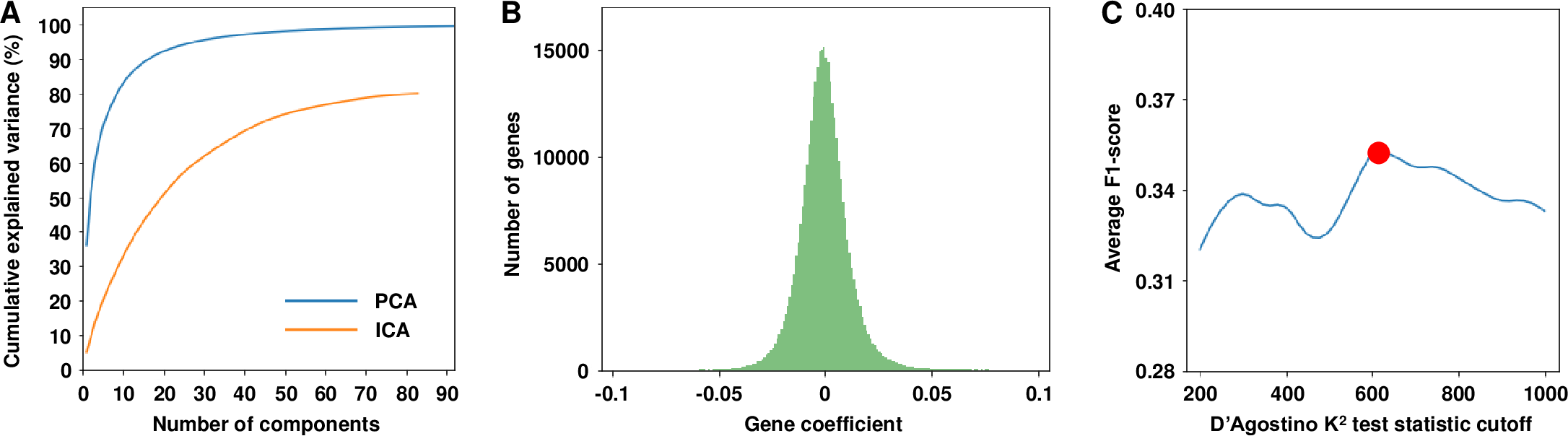

Supplement: S1 Fig — (A) Cumulative explained variance (CEV) for the transcriptome data of V. vulnificus was calculated by using principal component analysis (PCA, blue line) and independent component analysis (ICA, orange line). Using the 83 independent components, ICA reconstructed 80% of the total explained variance of the transcriptome data calculated by PCA. (B) A histogram of the gene coefficients in the entire independent components. Most of the gene coefficients were distributed around zero, and the gene coefficients in any independent component display a similar distribution. (C) Average F1-scores calculated under the varied D’Agostino K2 statistic cutoff ranging from 200 to 1,000 with an increment of 50. The optimal cutoff value was identified as 600 where the highest average F1-score was observed (shown in a red dot). (TIF) [file ppat.1011064.s001.tif]

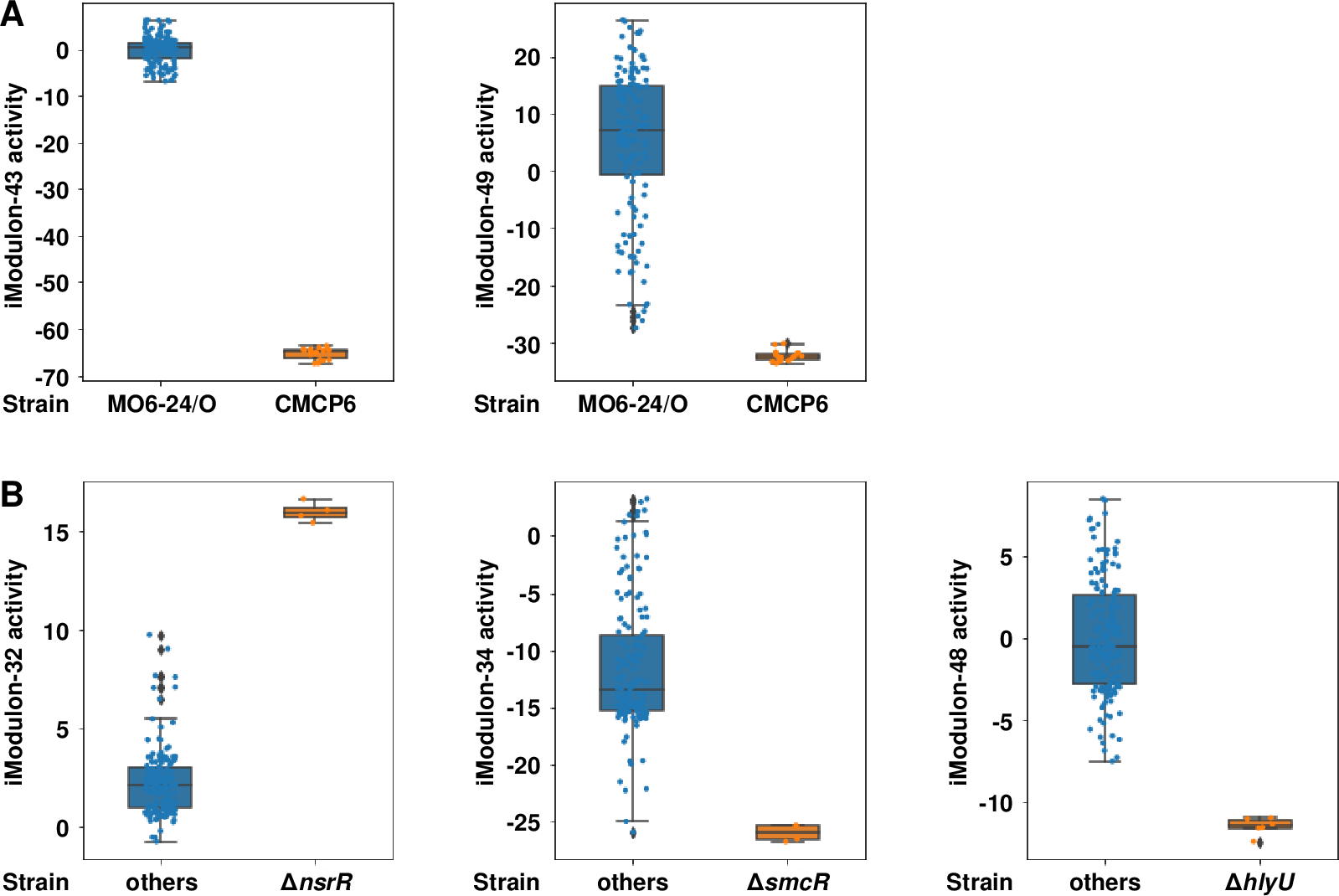

Supplement: S2 Fig — (A, B) Box plots of the iModulon activities under various conditions of the V. vulnificus cells. Box plot whiskers, box bounds, and the center line represent extrema, upper and lower quartiles, and the median value, respectively. MO6-24/O; CMCP6; ΔnsrR; ΔsmcR; ΔhlyU; others, transcriptome data from V. vulnificus MO6-24/O; V. vulnificus CMCP6; the nsrR mutant; the smcR mutant; the hlyU mutant; all other strains except the corresponding mutants, respectively. (TIF) [file ppat.1011064.s002.tif]

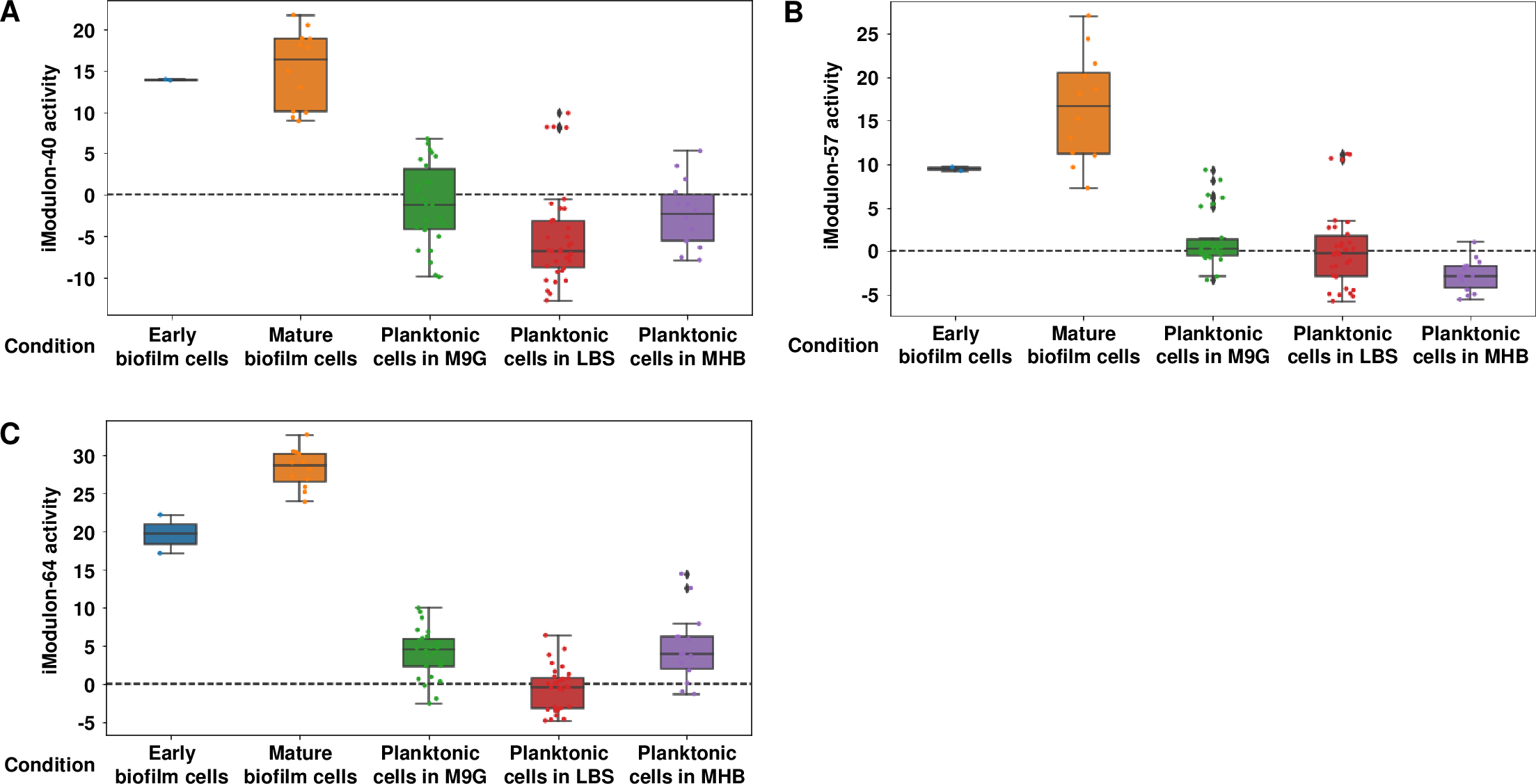

Supplement: S3 Fig — (A to C) Box plots of the iModulon activities under various conditions of biofilm and planktonic cells. Box plot whiskers, box bounds, and the center line represent extrema, upper and lower quartiles, and the median value, respectively. The gray dashed line represents zero. Early and mature biofilm cells indicate the biofilm cells incubated in VFMG for 1.5 h and 6~13 h, respectively. Among the various conditions of planktonic cells in the transcriptome data, conditions using M9G, LBS, and MHB as the growth medium were selected as the representatives. M9G, M9 minimal medium supplemented with 0.4% (w/v) glucose; LBS, Luria-Bertani (LB) medium supplemented with 2% (w/v) NaCl; MHB, Mueller-Hinton broth. (TIF) [file ppat.1011064.s003.tif]

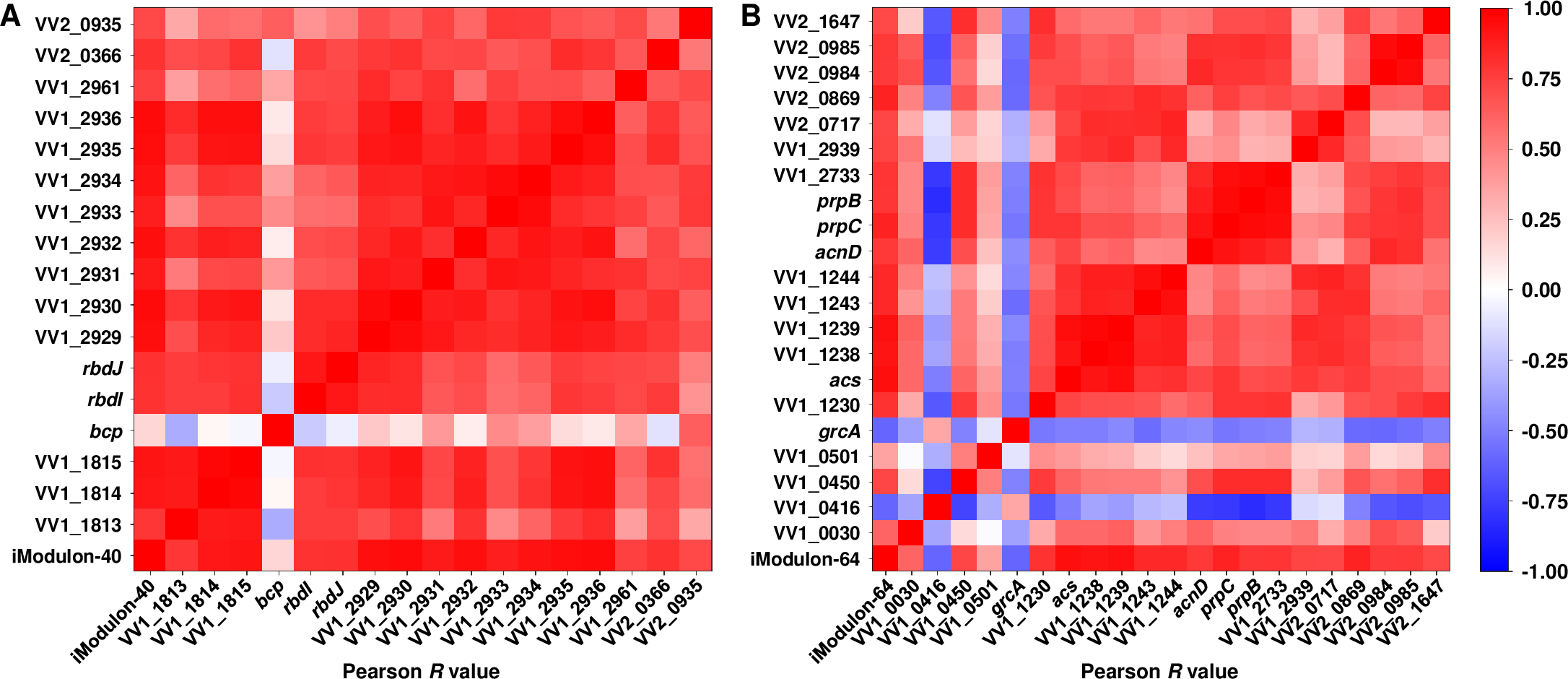

Supplement: S4 Fig — (A, B) Ordered correlation matrix. Colors indicate the Pearson R values between the activities of iModulon-40 (A) or iModulon-64 (B) and the expression levels of the element genes, respectively. Red and blue represent the strongest positive (+1) and negative (-1) correlation, respectively. (TIF) [file ppat.1011064.s004.tif]

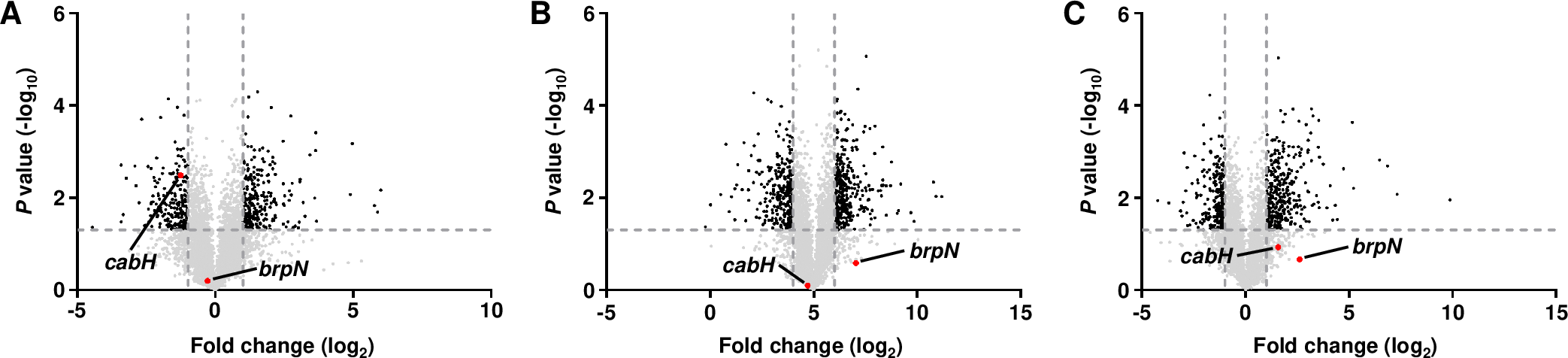

Supplement: S5 Fig — (A to C) Volcano plots from differential expression analysis between biofilm and planktonic cells. Total RNAs were isolated from the biofilm and planktonic V. vulnificus cells after incubation for 1.5 h (A), 6 h (B), and 10 h (C) in VFMG. Transcriptome analysis plotted the genes down-regulated or up-regulated in the biofilm cells (see S1 Methods for details). The black dots represent differentially expressed genes and the red dots represent cabH and brpN as indicated. The gray dashed lines indicate the cutoffs for differential expression of fold change > 2 and P value < 0.05. (TIF) [file ppat.1011064.s005.tif]

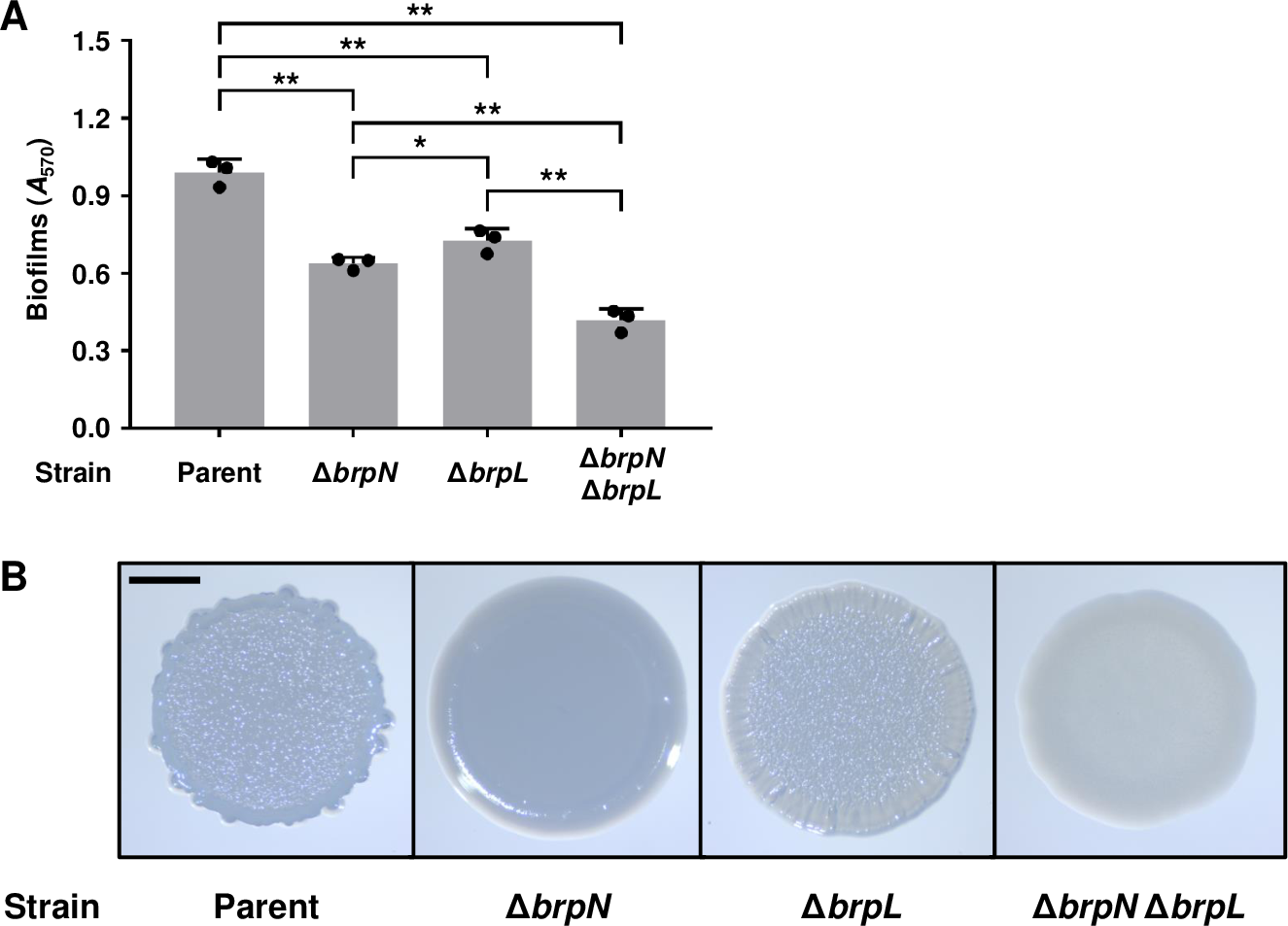

Supplement: S6 Fig — (A) Effects of brpN and brpL deletions on biofilm formation. For quantitative analysis of the biofilm, biofilms of the V. vulnificus strains were grown in VFMG supplemented with 0.01% arabinose in 96-well polystyrene microtiter plates for 24 h. Then supernatants were removed from the wells and the remaining biofilms were stained with 1% crystal violet. The crystal violet was eluted and its A570 was determined to quantify the biofilms (n = 3). Error bars represent the SD. Statistical significance was determined by the Student’s t test (*, P < 0.05; **, P < 0.005). (B) Effects of brpN and brpL deletions on colony morphology. The parent strain and mutants were spotted onto VFMG agar supplemented with 0.02% arabinose and incubated for 24 h. Each colony representing the mean rugosity from at least three independent experiments was visualized using a stereomicroscope (Stemi 305, Zeiss). All images are shown at the same scale, and a 1-mm scale bar is shown on the image of the parent strain. Parent, parent strain; ΔbrpN, brpN mutant; ΔbrpL, brpL mutant; ΔbrpN ΔbrpL, brpN brpL double mutant. (TIF) [file ppat.1011064.s006.tif]
