# Supplementary material for: Two novel genes identified by large-scale transcriptomic analysis are essential for biofilm and rugose colony development of Vibrio vulnificus
Source: PLoS Pathog. 2023 Jan 19;19(1):e1011064. doi: 10.1371/journal.ppat.1011064 (PMC9888727; doi:10.1371/journal.ppat.1011064)
Supplement: S1 Table — (DOCX) [file ppat.1011064.s007.docx]

**S1 Table. Overview of the iModulons identified by ICA.**

| iModulon | Number of genes | Biological function | iModulon category |
| --- | --- | --- | --- |
| iModulon-01 | 17 | Ribosomal RNA | Translation |
| iModulon-02 | 22 | RpoE stress response | Virulence and stress response |
| iModulon-03 | 23 | Fatty acid metabolism | Miscellaneous functions |
| iModulon-04 | 16 | Iron homeostasis | Metal homeostasis |
| iModulon-05 | 12 | Oxidative stress response | Virulence and stress response |
| iModulon-06 | 12 | Iron homeostasis | Metal homeostasis |
| iModulon-07 | 10 | Arginine biosynthesis | Amino acid and nucleotide biosynthesis |
| iModulon-08 | 7 | Galactose utilization | Carbon and nitrogen source utilization |
| iModulon-09 | 12 | Formate oxidation | Miscellaneous functions |
| iModulon-10 | 12 | Ribosomal RNA | Translation |
| iModulon-11 | 24 | Membrane constitution and modification | Structural components and signal transduction |
| iModulon-12 | 60 | Membrane constitution and modification | Structural components and signal transduction |
| iModulon-13 | 29 | Translation machinery | Translation |
| iModulon-14 | 15 | Peptide transport | Transport system |
| iModulon-15 | 18 | Sulfate assimilation and cysteine biosynthesis | Amino acid and nucleotide biosynthesis |
| iModulon-16 | 63 | Transfer RNA and ribosomal proteins | Translation |
| iModulon-17 | 17 | SOS response | Virulence and stress response |
| iModulon-18 | 22 | Chemotaxis | Structural components and signal transduction |
| iModulon-19 | 44 | Molecular chaperone | Miscellaneous functions |
| iModulon-20 | 30 | Hypochlorous acid stress response | Virulence and stress response |
| iModulon-21 | 6 | Transfer RNA | Translation |
| iModulon-22 | 5 | Lactate utilization | Carbon and nitrogen source utilization |
| iModulon-23 | 8 | PTS transport | Transport system |
| iModulon-24 | 12 | Oxidative stress response | Virulence and stress response |
| iModulon-25 | 43 | Unknown | Unknown |
| iModulon-26 | 9 | Biotin and thiamine biosynthesis | Miscellaneous functions |
| iModulon-27 | 66 | Various signal transductions | Structural components and signal transduction |
| iModulon-28 | 12 | Glycerol utilization | Carbon and nitrogen source utilization |
| iModulon-29 | 10 | Anaerobic metabolism | Miscellaneous functions |
| iModulon-30 | 18 | Copper resistance | Metal homeostasis |
| iModulon-31 | 8 | Ribosomal RNA | Translation |
| iModulon-32 | 8 | Accounts for *nsrR* knockout | Genomic difference |
| iModulon-33 | 4 | Pilus assembly | Structural components and signal transduction |
| iModulon-34 | 37 | Accounts for *smcR* knockout | Genomic difference |
| iModulon-35 | 8 | Multidrug efflux | Transport system |
| iModulon-36 | 12 | Nitrate and nitrite respiration | Carbon and nitrogen source utilization |
| iModulon-37 | 69 | Iron homeostasis | Metal homeostasis |
| iModulon-38 | 18 | Nitrogen assimilation | Carbon and nitrogen source utilization |
| iModulon-39 | 16 | Response to cell population density | Virulence and stress response |
| iModulon-40 | 17 | Related to biofilm formation | Miscellaneous functions |
| iModulon-41 | 18 | Pilus assembly | Structural components and signal transduction |
| iModulon-42 | 33 | Oxidative stress response | Virulence and stress response |
| iModulon-43 | 76 | Genomic difference between  MO6-24/O and CMCP6 strains | Genomic difference |
| iModulon-44 | 9 | Glycine betaine biosynthesis | Amino acid and nucleotide biosynthesis |
| iModulon-45 | 18 | Flagellar assembly | Structural components and signal transduction |
| iModulon-46 | 13 | Maltose utilization | Carbon and nitrogen source utilization |
| iModulon-47 | 5 | Unknown | Unknown |
| iModulon-48 | 17 | Accounts for *hlyU* knockout | Genomic difference |
| iModulon-49 | 92 | Genomic difference between  MO6-24/O and CMCP6 strains | Genomic difference |
| iModulon-50 | 4 | Electron transfer from cytochrome c to oxygen | Miscellaneous functions |
| iModulon-51 | 6 | Trimethylamine N-oxide respiration | Carbon and nitrogen source utilization |
| iModulon-52 | 20 | Glycerol-3-phosphate uptake | Carbon and nitrogen source utilization |
| iModulon-53 | 28 | Amino acid metabolism | Miscellaneous functions |
| iModulon-54 | 13 | Methionine biosynthesis | Amino acid and nucleotide biosynthesis |
| iModulon-55 | 50 | Various metabolisms | Miscellaneous functions |
| iModulon-56 | 16 | Purine biosynthesis | Amino acid and nucleotide biosynthesis |
| iModulon-57 | 14 | Biofilm formation | Virulence and stress response |
| iModulon-58 | 4 | Unknown | Unknown |
| iModulon-59 | 46 | Ribosomal proteins | Translation |
| iModulon-60 | 15 | Flagellin and chemotaxis | Structural components and signal transduction |
| iModulon-61 | 13 | Tryptophan, leucine, and glutamate biosynthesis | Amino acid and nucleotide biosynthesis |
| iModulon-62 | 11 | Unknown | Unknown |
| iModulon-63 | 47 | Various stress responses | Virulence and stress response |
| iModulon-64 | 21 | Central carbon metabolism | Carbon and nitrogen source utilization |
| iModulon-65 | 4 | Methionine biosynthesis | Amino acid and nucleotide biosynthesis |
| iModulon-66 | 8 | Biopolymer transport | Transport system |
| iModulon-67 | 8 | Phosphate transport | Transport system |
| iModulon-68 | 4 | Threonine biosynthesis | Amino acid and nucleotide biosynthesis |
| iModulon-69 | 19 | Unknown | Unknown |
| iModulon-70 | 8 | Prophage | Miscellaneous functions |
| iModulon-71 | 9 | Unknown | Unknown |
| iModulon-72 | 5 | Histidine biosynthesis | Amino acid and nucleotide biosynthesis |
