# Supplementary material for: Two novel genes identified by large-scale transcriptomic analysis are essential for biofilm and rugose colony development of Vibrio vulnificus
Source: PLoS Pathog. 2023 Jan 19;19(1):e1011064. doi: 10.1371/journal.ppat.1011064 (PMC9888727; doi:10.1371/journal.ppat.1011064)
Supplement: S2 Table — (DOCX) [file ppat.1011064.s008.docx]

**S2 Table. The element genes included in the iModulon-40.**

| Locus tag*^a^* | Gene*^b^* | Gene coefficient*^c^* | Annotation*^d^* |
| --- | --- | --- | --- |
| VV1_1813 | - | 0.1435 | LON peptidase substrate-binding domain-containing protein |
| VV1_1814 | - | 0.1699 | RNA polymerase sigma factor |
| VV1_1815 | - | 0.1745 | ChrR family anti-sigma-E factor |
| VV1_1910 | *bcp* | 0.1104 | thioredoxin-dependent thiol peroxidase |
| VV1_2666 | *rbdI* | 0.0763 | glycosyltransferase |
| VV1_2667 | *rbdJ* | 0.0851 | glycosyltransferase family 4 protein |
| VV1_2929 | - | 0.1708 | DUF3833 domain-containing protein |
| VV1_2930 | - | 0.1867 | chalcone isomerase family protein |
| VV1_2931 | - | 0.1805 | DUF2878 domain-containing protein |
| VV1_2932 | - | 0.2019 | cyclopropane-fatty-acyl-phospholipid synthase family protein |
| VV1_2933 | - | 0.2013 | DUF1365 family protein |
| VV1_2934 | - | 0.2096 | NAD(P)/FAD-dependent oxidoreductase |
| VV1_2935 | - | 0.2075 | SDR family oxidoreductase |
| VV1_2936 | - | 0.2087 | nuclear transport factor 2 family protein |
| VV1_2961 | - | 0.0836 | DUF2982 domain-containing protein |
| VV2_0366 | - | 0.0900 | DUF4174 domain-containing protein |
| VV2_0935 | - | 0.1003 | cryptochrome/photolyase family protein |

*^a, b,^* ^and^ *^d^* Locus tags, gene names, and annotations are based on the *V. vulnificus* CMCP6 genome (GenBank accession numbers: AE016795.3 and AE016796.2).

*^b^* Unnamed genes are described as ‘-’.

*^c^* Gene coefficients of the element genes in the iModulon are based on S3 Dataset.
