# Supplementary material for: Two novel genes identified by large-scale transcriptomic analysis are essential for biofilm and rugose colony development of Vibrio vulnificus
Source: PLoS Pathog. 2023 Jan 19;19(1):e1011064. doi: 10.1371/journal.ppat.1011064 (PMC9888727; doi:10.1371/journal.ppat.1011064)
Supplement: S3 Table — (DOCX) [file ppat.1011064.s009.docx]

**S3 Table. The element genes included in the iModulon-64.**

| Locus tag*^a^* | Gene*^b^* | Gene coefficient*^c^* | Annotation*^d^* |
| --- | --- | --- | --- |
| VV1_0030 | - | 0.1071 | TRAP transporter substrate-binding protein |
| VV1_0416 | - | -0.0963 | GPR1/FUN34/YaaH family transporter |
| VV1_0450 | - | 0.1022 | malate synthase A |
| VV1_0501 | - | 0.0827 | carbon starvation protein A |
| VV1_0542 | *grcA* | -0.0864 | autonomous glycyl radical cofactor GrcA |
| VV1_1230 | - | 0.0942 | methyl-accepting chemotaxis protein |
| VV1_1237 | *acs* | 0.2190 | acetate--CoA ligase |
| VV1_1238 | - | 0.1586 | 3'-5' exonuclease |
| VV1_1239 | - | 0.1848 | DUF294 nucleotidyltransferase-like domain-containing protein |
| VV1_1243 | - | 0.0971 | cation acetate symporter |
| VV1_1244 | - | 0.1180 | DUF4212 domain-containing protein |
| VV1_2730 | *acnD* | 0.0855 | Fe/S-dependent 2-methylisocitrate dehydratase AcnD |
| VV1_2731 | *prpC* | 0.0989 | 2-methylcitrate synthase |
| VV1_2732 | *prpB* | 0.0997 | methylisocitrate lyase |
| VV1_2733 | - | 0.1067 | GntR family transcriptional regulator |
| VV1_2939 | - | 0.1604 | hypothetical protein |
| VV2_0717 | - | 0.1024 | hypothetical protein |
| VV2_0869 | - | 0.0871 | aldehyde dehydrogenase |
| VV2_0984 | - | 0.0818 | TRAP transporter permease |
| VV2_0985 | - | 0.1032 | TAXI family TRAP transporter solute-binding subunit |
| VV2_1647 | - | 0.0849 | malate synthase |

*^a, b,^* ^and^ *^d^* Locus tags, gene names, and annotations are based on the *V. vulnificus* CMCP6 genome (GenBank accession numbers: AE016795.3 and AE016796.2).

*^b^* Unnamed genes are described as ‘-’.

*^c^* Gene coefficients of the element genes in the iModulon are based on S3 Dataset.
