# Supplementary material for: Two novel genes identified by large-scale transcriptomic analysis are essential for biofilm and rugose colony development of Vibrio vulnificus
Source: PLoS Pathog. 2023 Jan 19;19(1):e1011064. doi: 10.1371/journal.ppat.1011064 (PMC9888727; doi:10.1371/journal.ppat.1011064)
Supplement: S4 Table — (DOCX) [file ppat.1011064.s010.docx]

**S4 Table. The GC contents of *brpN*, *brpLG*, the *brp* locus, and chromosome II.**

| DNA sequence*^a^* | Locus tag*^b^* | GC content*^c^* (%) |
| --- | --- | --- |
| *brpN* | VV2_1694 | 33 |
| *brpLG* | VV2_1626-1627 | 42 |
| *brp* locus | VV2_1574-1582 | 43 |
| chromosome II | - | 47 |

*^a^* ^and^ *^b^* Gene names and locus tags are based on the *V. vulnificus* CMCP6 genome chromosome II (GenBank accession number: AE016796.2).

*^c^* The GC contents are represented as the GC percent of the corresponding DNA sequence.
