# Supplementary material for: Two novel genes identified by large-scale transcriptomic analysis are essential for biofilm and rugose colony development of Vibrio vulnificus
Source: PLoS Pathog. 2023 Jan 19;19(1):e1011064. doi: 10.1371/journal.ppat.1011064 (PMC9888727; doi:10.1371/journal.ppat.1011064)
Supplement: S5 Table — (DOCX) [file ppat.1011064.s011.docx]

| Strain or plasmid | Relevant characteristics*^a^* | Reference of source |
| --- | --- | --- |
| Bacterial strains | | |
| *V. vulnificus* | | |
| JN111 | CMCP6 with P_BAD_-*dcpA*, parent strain | [1] |
| JN161D | JN111 with Δ*brpT* | [2] |
| SH173 | JN111 with Δ*cabH* (VV1_3061) | This study |
| HJ213 | JN111 with Δ*brpN* (VV2_1694) | This study |
| YM112D | JN111 with Δ*cabA* | [1] |
| SH201 | JN111 with Δ*brpL* | [3] |
| HJ214 | JN111 with Δ*brpN* Δ*brpL* | This study |
| *E. coli* | | |
| S17-1 λ*pir* | λ-*pir* lysogen; *thi pro hsdR hsdM*^+^ *recA* RP4-2 Tc::Mu-Km::Tn7;Tp^r^ Sm^r^ ; host for π-requiring plasmids | [4] |
| BL21 (DE3) | *F*^-^, *ompT*, *hsdS* (r_B_^-^, m_B_^-^), *gal dcm* (DE3) | Laboratory collection |
| Plasmids | | |
| pDM4 | R6K γ *ori sacB*; suicide vector; *oriT* of RP4; Cm^r^ | [5] |
| pSH1703 | pDM4 with Δ*cabH* (VV1_3061); Cm^r^ | This study |
| pHJ2103 | pDM4 with Δ*brpN* (VV2_1694); Cm^r^ | This study |
| pSH1819 | pET-28a(+) with *brpT*; Km^r^ | [2] |
| pJK1113 | pKS1101 with *nptI*; Ap^r^ Km^r^ | [6] |
| pHJ2202 | pJK1113 with *cabH*; Ap^r^ Km^r^ | This study |
| pHJ2203 | pJK1113 with *brpN*; Ap^r^ Km^r^ | This study |

**S5 Table. Bacterial strains and plasmids used in this study.**

*^a^* Tp^r^, trimethoprim-resistant; Sm^r^, streptomycin-resistant; Cm^r^, chloramphenicol-resistant; Km^r^, kanamycin-resistant; Ap^r^, ampicillin-resistant.

# References

1. Park JH, Jo Y, Jang SY, Kwon H, Irie Y, Parsek MR, et al. The *cabABC* Operon Essential for Biofilm and Rugose Colony Development in *Vibrio vulnificus*. Plos Pathog. 2015;11(10). doi: 10.1371/journal.ppat.1005252. PMID: 26406498

2. Hwang SH, Park JH, Lee B, Choi SH. A Regulatory Network Controls *cabABC* Expression Leading to Biofilm and Rugose Colony Development in *Vibrio vulnificus*. Front Microbiol. 2020;10. doi: 10.3389/fmicb.2019.03063. PMID: 32010109

3. Hwang SH, Im H, Choi SH. A Master Regulator BrpR Coordinates the Expression of Multiple Loci for Robust Biofilm and Rugose Colony Development in *Vibrio vulnificus*. Front Microbiol. 2021;12. doi: 10.3389/fmicb.2021.679854. PMID: 34248894

4. Simon R, Priefer U, Puhler A. A Broad Host Range Mobilization System for Invivo Genetic-Engineering - Transposon Mutagenesis in Gram-Negative Bacteria. Bio-Technol. 1983;1(9):784-91. doi: 10.1038/nbt1183-784.

5. Milton DL, OToole R, Horstedt P, WolfWatz H. Flagellin A is essential for the virulence of *Vibrio anguillarum*. J Bacteriol. 1996;178(5):1310-9. doi: DOI 10.1128/jb.178.5.1310-1319.1996. PMID: 8631707

6. Lim JG, Bang YJ, Choi SH. Characterization of the *Vibrio vulnificus* 1-Cys Peroxiredoxin Prx3 and Regulation of Its Expression by the Fe-S Cluster Regulator IscR in Response to Oxidative Stress and Iron Starvation. J Biol Chem. 2014;289(52):36263-74. doi: 10.1074/jbc.M114.611020. PMID: 25398878
