# Supplementary material for: Two novel genes identified by large-scale transcriptomic analysis are essential for biofilm and rugose colony development of Vibrio vulnificus
Source: PLoS Pathog. 2023 Jan 19;19(1):e1011064. doi: 10.1371/journal.ppat.1011064 (PMC9888727; doi:10.1371/journal.ppat.1011064)
Supplement: S6 Table — (DOCX) [file ppat.1011064.s012.docx]

**S6 Table. Oligonucleotides used in this study.**

| Oligonucleotide | Oligonucleotide sequence (5’ →3’)*^a^* | Use |
| --- | --- | --- |
| For mutant construction | | |
| CABH01_F | GAGCTCAGGTTACCCGCATGAACGTGACCATCGATGCG | Deletion of *cabH* ORF |
| CABH01_R | AATCAAACAGCGTTGTTCCAGACATAGATTATTCC |  |
| CABH02_F | TGGAACAACGCTGTTTGATTTTGCTTGATAAGTGC |  |
| CABH02_R | CGACCCTCGAGTACGCGTCACACTGTTGTGCCGTGACC |  |
| BRPN01_F | CTCAGGTTACCCGCATGGTACAGCACTTAGGAGTC | Deletion of *brpN* ORF |
| BRPN01_R | GGTGATAATCCACGGATGCAGCAAGGCAGTACAACC |  |
| BRPN02_F | GGTTGTACTGCCTTGCTGCATCCGTGGATTATCACC |  |
| BRPN02_R | CTCGAGTACGCGTCAGTATTGAACAGGCCAATG |  |
| For mutant complementation | | |
| CABH03_F | GGCTAGCAGGAGGAATTCACATGTCTGGAACAACGGTTG | Amplification of *cabH* ORF |
| CABH03_R | AAAACAGCCAAGCTTGCATGAGTGACGATACTCGCACTTATC |  |
| BRPN03_F | GGCTAGCAGGAGGAATTCACATGTTCATTCAATCGATACACAAC | Amplification of *brpN* ORF |
| BRPN03_R | AAAACAGCCAAGCTTGCATGGTATGGAATGATGGCGAAAG |  |
| For EMSA | | |
| CABHUP_F | GTCTTACCAAGCTAGACCCGT | Amplification of *cabH* upstream region |
| CABHUP_R | TGATGATATTGACTGCAAGCGT |  |
| BRPNUP_F | GCATCAGTCTAAACACCGCAC | Amplification of *brpN* upstream region |
| BRPNUP_R | TGGCGATGGCTCTAAAGTTGT |  |
| For qRT-PCR | | |
| BRPT_qRT_F | GAAGCTGTGTCGCGGGATTG | Quantification of *brpT* expression |
| BRPT_qRT_R | TGTGGCTCTTCCTTCTTCGCTC |  |
| CABH_qRT_F | ATAAGTTCGTCGGCTTCGGG | Quantification of *cabH* expression |
| CABH_qRT_R | CCGCCAAAGAGGGTGTCATT |  |
| BRPN_qRT_F | GCTCAACCGCAGATTATGGA | Quantification of *brpN* expression |
| BRPN_qRT_R | ATGTAGTGGAGCGCAAAGGA |  |
| RRSH_qRT_F | TAACGCTCGCACCCTCCGTA | Quantification of *rrsH* expression |
| RRSH_qRT_R | CATGCCGCGTGTGTGAAGAA |  |

*^a^* Regions of oligonucleotides not complementary to the corresponding genes are underlined.
