# Supplementary material for: Two novel genes identified by large-scale transcriptomic analysis are essential for biofilm and rugose colony development of Vibrio vulnificus
Source: PLoS Pathog. 2023 Jan 19;19(1):e1011064. doi: 10.1371/journal.ppat.1011064 (PMC9888727; doi:10.1371/journal.ppat.1011064)
Supplement: S1 Methods — (DOCX) [file ppat.1011064.s019.docx]

**S1 Methods. Differential expression analysis of the genes in biofilm and planktonic cells.**

Raw sequencing reads of RNA-seq data were retrieved from the Sequence Read Archive (SRA) database at NCBI (SRA accession number SRR20681973 to SRR20681984, listed in S1 Dataset). The reads were mapped to the reference genome of *Vibrio vulnificus* (GenBank accession numbers CP002469.1 for chromosome I and CP002470.1 for chromosome II) using CLRNASeq software (Chunlab, Seoul, South Korea). The expression level of genes was determined by counting the number of reads per kilobase per million mapped reads (RPKM), and quantile normalization was conducted on the RPKM values using CLC Genomics Workbench 6.5 (CLC Bio, Aarhus, Denmark). After normalization, fold changes of RPKM values and their significances (*P* values) were assigned to the genes using CLC Genomics Workbench 6.5.
