# Supplementary material for: Two novel genes identified by large-scale transcriptomic analysis are essential for biofilm and rugose colony development of Vibrio vulnificus
Source: PLoS Pathog. 2023 Jan 19;19(1):e1011064. doi: 10.1371/journal.ppat.1011064 (PMC9888727; doi:10.1371/journal.ppat.1011064)
Supplement: Attachment — Submitted filename: 221130_revision_CoverLetter.pdf [file ppat.1011064.s020.pdf]

Manuscript No: Revised version of PPATHOGENS-D-22-01592 November 30, 2022

Prof. Matthew Parsek, Associate Editor
Prof. Karla Satchell, Section Editor
Prof. Kasturi Haldar, Editor-in-Chief
Prof. Michael Malim, Editor-in-Chief

PLoS Pathogens
1265 Battery Street, Suite 200
San Francisco, CA 94111
United States

Dear editors,

We greatly appreciate the efforts and comments from you and both reviewers on our manuscript "**Two novel genes identified by large-scale transcriptomic analysis are essential for biofilm and rugose colony development of *Vibrio vulnificus***". We have performed additional data analyses and experiments, and rewritten parts of the manuscript to comply with the reviewers' comments. Particularly, as Reviewer #1 requested, we have provided expanded information on the ICA workflow and the resulting iModulons (Figs 1 and 2). We have also shown the differences between the strains more clearly by quantitative analysis of the biofilms in the test tubes as Reviewer #2 suggested (Fig 8C and 8D).

Our point-by-point responses to the reviewers' comments are presented below and all changes to the manuscript are highlighted in yellow. We acknowledge that all the reviewers' comments are highly valuable and have led us to improve the paper. We hope that these amendments and added information will suffice you and the reviewers for publication of the revised manuscript in PLoS Pathogens.

Thank you again for your kind consideration, and we look forward to hearing from you again.

Sincerely yours,

Sang Ho Choi, Corresponding author
National Research Laboratory of Molecular Microbiology and Toxicology,
Department of Agricultural Biotechnology, Seoul National University,
Seoul 08826, Republic of Korea
Phone: +82-2-880-4857, Fax: +82-2-873-5095
Email: choish@snu.ac.kr

**Point-by-point response (Manuscript No: Revised version of PPATHOGENS-D-22-01592)**

**Reviewer #1, Prof. Joe J. Harrison**

**Summary**

In this manuscript, Lee and colleagues use a machine learning algorithm to identify groups of co-regulated genes, termed iModulons, in the *Vibrio vulnificus* genome linked to biofilm formation. The bioinformatic deep-dive was executed by using independent component analysis (ICA) to re-analyze hundreds of transcriptomic datasets publicly available through NCBI. This approach was validated by identifying 2 additional genes, *cabH* and *brpN*, that are part of the BrpT regulon, which regulates extracellular polysaccharide production and biofilm formation in *V. vulnificus*.
The manuscript is technically sound. The approach to genetic analysis for *cabH* and *brpN* is thorough – including phenotyping of mutants, complementation analysis, and demonstration of BrpT-dependent gene expression using EMSAs, which are not trivial. Taken together, this comprehensive approach provides confidence in the genetic linkages and provides proof-of-principle for the bioinformatic approach. I enjoyed reading this article.

**Major Issue 1.**

Perhaps the most interesting thing in the paper is the analysis of transcriptional regulatory networks in *V. vulnificus* using ICA to identify iModulons. However, relatively little information about this machine learning approach is presented in the manuscript. The descriptions leave me wanting to know more. I suspect it will be the same for other readers. Could the authors provide an expanded description of these methods and the "Modulome" workflow? This is exciting, new technology.

**Response**

This is one of the most valuable suggestions. To address the Reviewer's suggestion, we have included the graphical illustration and description of the ICA workflow in the revised manuscript (Fig 1 and lines 103-106). In addition, some of the contents previously included in Fig 10 have been moved to Fig 1 to explain the overall ICA workflow comprehensively.

**Major Issue 2.**

There is a lot of information yielded by the Modulome workflow that is glossed over in the manuscript. Could the authors consider providing a display item that summarizes some of the insights into *V. vulnificus* regulatory networks provided by this analysis? I realize that this is not a small ask – but it comes across as an omission. As a microbiologist, I want to know more about what machine learning can reveal about gene expression in this bacterium.

**Response**

We appreciate the comment. As the Reviewer noted, plenty of information can be obtained by analyzing iModulons and their element genes. We have investigated the element genes and annotated the iModulons with specific biological functions (S1 Table). Then, we have grouped the iModulons into 10 categories according to their biological functions (lines 132-141, S1 Table, and S2 Fig). The iModulons grouped into the categories have been displayed in Fig 2.
As the previous papers analyzing the bacterial transcriptome data through ICA, iModulons could be annotated with regulators controlling their element genes (Sastry *et al*., 2019; Rajput *et al*., 2022). However, since there is limited information on *V. vulnificus* regulatory networks, many iModulons could not be reliably annotated with regulators. Thus, we have annotated the iModulons with the biological functions, instead.

**Major Issue 3.**
Can the authors add their datasets to iModulonDB? See (https://academic.oup.com/nar/article/49/D1/D112/5921295).

**Response**
Yes, we would like to add our datasets to iModulonDB. Upon being published, our datasets will be submitted to iModulonDB.

**Minor Issue 1.**
In all bar graphs, could the authors please show their individual datum points so that the readers can evaluate data distributions for themselves.

**Response**
We appreciate the suggestion. To enable readers to assess the data distributions, each datum point has been displayed in the bar graphs (Figs 5, 6, 8, 9, and S6).

**Minor Issue 2.**
While it is possible that rendering in Editorial Manager may have affected figure quality, it is very difficult to see rugose colony morphology in the figures. Could the authors double check the image quality, or alternatively, take better photos so that rugosity is easily visible to the reader.

**Response**
We thank for the careful comment. We double-checked the quality of all figures and confirmed that the colony rugosity can be more clearly identified in the image files (‘Fig_7.tif’ and ‘S6_Fig.tif’) rather than in the word file.

**<u>Reviewer #2, Prof. Kyle A. Floyd</u>**

**Summary**
The ability to form multicellular bacterial communities, or biofilms, is predominant among bacterial species. Biofilm formation provides protection to the bacterial community and allows opportunities for nutrient acquisition/concentration and horizontal gene transfer. This protected communal lifestyle allows for the survival and persistence of bacteria in nature. Among biofilm-forming bacteria, are naturally occurring aquatic *Vibrio* species. *Vibrionaceae* species such as *V. cholerae*, *V. parahaemolyticus*, and *V. vulnificus* are facultative human pathogens, with significant and devastating impacts to public health. Biofilm formation is key to the environmental survival and persistence of *Vibrio* species and can impart a substantial role in host pathogenesis and disease. In this work, Lee et al. performed an in-depth analysis of transcriptome data comparing planktonic and biofilm modalities within the seafood-borne pathogen *V. vulnificus*. The objective was to use an unbiased machine learning algorithm, Independent Component Analysis (ICA), to identify genetic regulons (iModulons) related to biofilm and rugose colony formation in *V. vulnificus*.
The authors identified several biofilm-associated iModulons; one of which contained genes modulated by the known *V. vulnificus* biofilm transcriptional regulator, BrpT. Along with genes known to be regulated by BrpT, the authors identify two novel BrpT-regulated genes of unknown function: VV1_3061 (*cabH*) and VV2_1694 (*brpN*). The authors next validate these two genes as BrpT-regulated through qRT-PCR, and predict their function based on structural homology to known *V. vulnificus* genes. The authors then beautifully demonstrate that CabH appears to be involved in surface-attachment, and BrpN appears to be involved in production or secretion of exopolysaccharide. Overall, I feel that the quality of work and data analysis is strong, and that the results match the interpretation developed by the authors.

I feel that the work presented here is novel and represents a very significant advancement for the study of biofilm regulation in bacteria. The authors present a very well written and detailed step-by-step procedure for the computational identification of previously unknown genes involved in *V. vulnificus* biofilm formation, and for molecular verification of results predicted by computational analysis. Not only is this work significant for the study of both *V. vulnificus* and biofilm biology, but it is also a significant step forward in development and utilization of unbiased data analysis methods for the interpretation of large transcriptome datasets. I do feel that this work is acceptable for publication in PLoS Pathogens and would be of key interest to its audience. Therefore, with consideration of the minor revisions outlined below, I would recommend publication of this work.

**Minor Issue 1.**

- Figure 3A-B: How many biological replicates were used for qRT-PCR analysis? Please state in the Figure Legend or Materials and Methods.
- Figure 3C-D: How many independent times were the EMSA analyses performed? Provided gel images are representative of how many assays? Please state in the Figure Legend or Materials and Methods.
- Figure 4: How many biological replicates were used for the crystal violet staining biofilm assay? Please state in the Figure Legend or Materials and Methods.
- Figure 6: How many biological replicates were used for the crystal violet staining biofilm assay? Please state in the Figure Legend or Materials and Methods.

**Response**

We appreciate the valuable comments and fine suggestions. We have stated the number of biological replicates used in each experiment in the figure legends (Figs 5, 6, 8, 9, and S6). Also, we have specified the number of independent trials for the gel images in the figure legends, too (Figs 5 and 9).

**Minor Issue 2.**

- Figure 6: While I agree with the conclusions of the authors that CabH appears to be involved in surface attachment, and BrpN impacts biofilm formation independent of attachment, I do feel that this assertion could be strengthened by quantification of the crystal violet used for staining here after washing of the biofilm. This would not only allow for quantification of the differences between the isogenic parent strain and each mutant tested but would also allow for determination of more subtle differences between the strains.

This assertion could also be strengthened by the microscopic analysis of the ability of the *cabH* mutant to attach to surfaces. Is the mutant impaired in initial attachment, or can it initially attach to a surface and the impairment is in the long-term ability to stay on a surface (given that the biofilms are analyzed at the mature state od 24H)? While this analysis may not be required for this publication, I do believe it is something that the authors should consider.

**Response**

We thank the thoughtful suggestions. To address the suggestions, we have first quantified the biofilms of the *cabH*, *brpN*, and *cabA* mutants in the test tubes (lines 507-508 and Fig 8C and 8D). As expected, the mutants showed reduced biofilm levels compared with the parent strain in both glass and polystyrene test tubes (Fig 8). Among the mutants, the *cabH* mutant (Δ*cabH*) specifically showed more decreased biofilm formation in smooth-surfaced glass tubes than in polystyrene tubes (Fig 8). The results suggest that Δ*cabH* with impaired surface attachment has more difficulties in attaching to smoother materials (lines 278-281).

As the reviewer pointed, the poor surface attachment of Δ*cabH* was possibly resulted from either the impaired initial attachment or damage in the long-term ability to stay on a surface. The attachment ability shown in Fig 8A and 8B could not be visually differentiated in the initial stage due to the tiny amount of biofilm. Instead, the tiny amount of the biofilm in the initial stage was spectrophotometrically quantified. As shown below in Fig R1A, the parent strain and Δ*cabH* produced similar levels of biofilm in the initial stage, suggesting that Δ*cabH* is not defective in initial attachment. Therefore, a microscopic

analysis was performed to find clues about the long-term ability to stay on a surface. As shown below in Fig R1B, Δ*cabH* produced less amount of extracellular matrix, which contributes to the bacterial long-term ability to stay on a surface (Flemming et al., 2010), than the parent strain. The results propose that CabH may be involved in the long-term ability to stay on a surface rather than in initial attachment, by being involved in matrix development. However, more research is required to verify this proposal more clearly. Therefore, we decided to share Fig R1 only with the reviewers without including it in the revised manuscript. We deeply appreciate the insightful comment again.

**Changes in the manuscript which are not mentioned in Point-by-point response**

One of the authors, Duhyun Ko, has changed her affiliation, so we have included her current address in the title page (lines 8 and 15-16).

We have rewritten several sentences in Author Summary to make it easier to understand (lines 43-49).

We have included a sentence 'The stained biofilms were then washed with vibration in PBS to remove loosely attached cells.' in figure legends of Fig 8 for further explanation (lines 286-287).

We have modified the phrase 'after the PBS wash' to 'after being washed with PBS' (lines 289 and 507).

We have added the word 'structurally' to clarify the meaning (line 380).

We have changed the expression 'activates *cabABC* expression and represses *brpT* expression' to 'activates the expression of *cabABC* and represses the expression of *brpT*' in figure legends of Fig 10 (lines 391).

We have improved a sentence, but it still means the same thing (lines 424-427).

We have slightly modified the title of S2, S3, and S4 datasets (lines 770-772).

To clearly distinguish the two proteins, we have colored the protein names green and magenta in Fig 4B and 4C.

**References**

1. Sastry AV, Gao Y, Szubin R, Hefner Y, Xu SB, Kim D, et al. The *Escherichia coli* transcriptome mostly consists of independently regulated modules. Nat Commun. 2019;10. doi: 10.1038/s41467-019-13483-w. PMID: 31797920
2. Rajput A, Tsunemoto H, Sastry AV, Szubin R, Rychel K, Sugie J, et al. Machine learning from *Pseudomonas aeruginosa* transcriptomes identifies independently modulated sets of genes associated with known transcriptional regulators. Nucleic Acids Res. 2022;50(7):3658-72. doi: 10.1093/nar/gkac187. PMID: 35357493
3. Flemming HC, Wingender J. The biofilm matrix. Nat Rev Microbiol. 2010;8(9):623-33. doi: 10.1038/nrmicro2415. PMID: 20676145

**Additional Data (for reviewers only)**

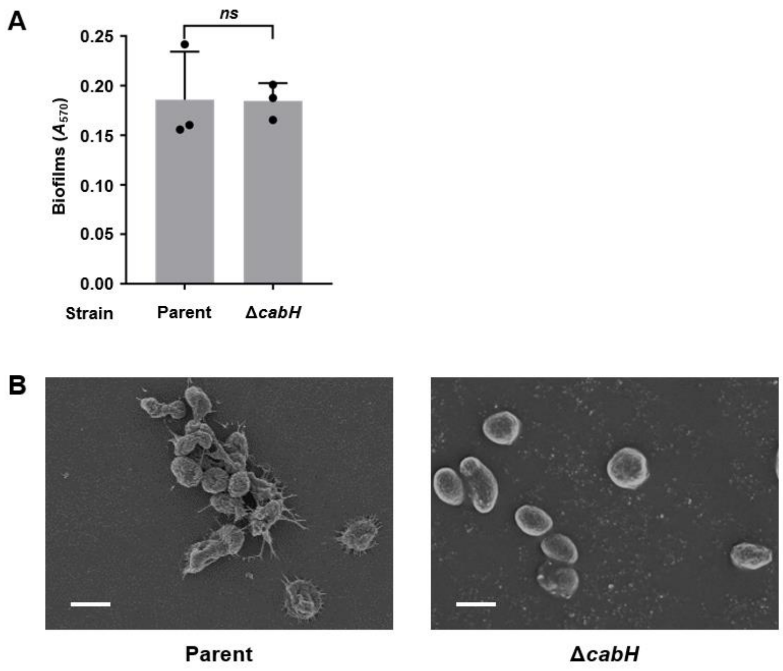


**Fig R1. Biofilm formation and structures of the *cabH* mutant.** (A) To quantify the biofilms of the *V. vulnificus* strains, each well of the 96-well polystyrene microtiter plates was inoculated with 200 µl of culture diluted to an $A_{600}$ of 0.05 in VFMG supplemented with 0.01% arabinose and then incubated for 2 h. Then supernatants were removed from the tubes and the remaining biofilms were stained with 1% crystal violet. The crystal violet was eluted and its absorbance at 570 nm ($A_{570}$) was determined to quantify the biofilms ($n$ = 3). Error bars represent the SD. Statistical significance was determined by the Student's $t$ test (*ns*, not significant). (B) For structural analyses, biofilms of the strains were formed in flow cell chambers. Glass coverslips were stuck to polycarbonate flow cells with individual channel dimensions of 1 × 4 × 40 mm. Each flow cell was inoculated with 100 µl of the culture diluted to $A_{600}$ of 0.1, and inverted to allow bacteria to attach to the coverslip for 1 h without flow. Then VFMG supplemented with 0.01% arabinose was flowed at a constant rate of 8 ml/h using a Minipuls Evolution peristaltic pump (Gilson, Villiers-le-Bel, France) to grow biofilms for 3 days. Biofilms were fixed, dehydrated, coated with platinum, and visualized using SEM (Supra 55VP, Zeiss) at 10,000 × magnification. 1-µm scale bars are shown on each image. Parent, parent strain; Δ*cabH*, *cabH* mutant.
